# On Truthful Introductions

**DOI:** 10.15694/mep.2018.0000054.1

**Published:** 2018-03-06

**Authors:** Richard Prayson, Elizabeth E. O'Toole

**Affiliations:** 1 Cleveland Clinic; 2 MetroHealth Medical Center

**Keywords:** Communication, Professionalism, Truthfulness in communication

## Abstract

This article was migrated. The article was marked as recommended.

None

## Personal View Paper

I recently had a second year medical student come pay me visit. I work in the Student Affairs office and routinely have students stop by for a variety of issues or sometimes just to chat. This student’s visit ostensibly seemed to be a chat until he worked up the nerve to ask the question, “Is it ever right for a medical student to introduce themselves as a doctor?”

“What do you mean?”

“Well, I know we are not supposed to introduce ourselves to real patients as doctors but we were told to introduce ourselves as a doctor as part of an OSCE we had the other day. It just seemed a bit weird.”

Upon further inquiring of the person in change of the exercise, I discovered that the OSCE was focused on interviewing a patient. The topic of the interview was a difficult conversation with a patient that normally a student would not be asked to have. The rationale was that the student should put her or himself in the shoes of a physician, even to the extent of introducing her or himself as a physician, in order to facilitate the discussion and perhaps put the student more at ease. This student, not particularly a fan of role plays and OSCEs to start with, found he was more self-conscious than usual in pretending he was someone that he was not by introducing himself as someone he was not. He became distracted by it and felt it impacted his performance in the exercise.

This subject of introducing oneself as a physician while still a medical student has been fodder for discussion in the literature for decades. Almost everyone agrees that we, either as physicians or physicians in training, should always introduce ourselves to patients ( Beatty et al 1995). It is the socially proper thing to do and it is important in establishing rapport and trust with our patients, the foundation of a therapeutic relationship ( Granger 2013). The corollary of this would be that the introduction should be truthful. In a 1992 survey of third and fourth year medical students, Beatty and Lewis found that 5% of students who responded to the survey routinely introduced themselves as “doctor”; all responders admitted that they had experienced being introduced as doctors by hospital staff, and only 42% of these students corrected this information with their patients ( Beatty et al. 1995).

Some students who play along with such inaccurate introductions likely feel a bit uncomfortable with the misrepresentation but have concerns that correcting the matter might interfere with patient rounds or somehow make their staff look badly in the patient’s eyes. There is also student concern that patients may refuse to have students participate in their care, if patients know they are a student, potentially depriving them of a learning opportunity, not to mention the embarrassment of being told by a patient that one is not welcome ( Marracino et al. 1998). There is also the concern that patients may not be willing to share important or embarrassing health information with a student that they otherwise might be willing to share with a physician. All of those who are physicians remember situations in which this occurred as students and the sense of failure and embarrassment that accompanied it. Students may feel that using the title “doctor” makes them a more legitimate member of the health care team and that the patient’s right to know the student’s identity is not that terribly important anyway ( Marracino et al. 1998). Some may even believe that there are times when not telling the patient the truth is acceptable or inconsequential ( Marracino et al, 1998).

The particular reason that was cited by the faculty in charge of the OSCE exercise was that students perform best when they think of themselves as a doctor. And after all, this was a role play and did not involve a real patient, so no harm, no foul. The student-patient relationship, as is the doctor-patient relationship, is predicated on trust; the relationship is undermined by lying ( Horn 1985). Pretending otherwise, even in a mock or simulated situation such as a role play or OSCE, serves no purpose and potentially sends the wrong message to a student. Being a student also affords the student more latitude to take more time and fumble around a bit in trying to learn the art of history taking and of the physical exam without feeling like they have to live up to the standard of a physician; patients expect more from their physicians. One can potentially create a situation or foster an environment in which the student is fearful of being discovered as an intellectual fraud and doing harm in the high stakes endeavor of taking care of sick patients; such feelings have been referred to as imposter syndrome and have been associated with burnout among medical students ( Villwock et al. 2016).

To quote Benjamin Mays, a minister and former president of Morehouse College, “Honest communication is built on trust and integrity and upon respect of the one for the other.” I believe the student who visited me was correct to be concerned and this serves as a reminder that we need to be constantly vigilant and careful and mindful on how we teach. Medical education has a commitment to creating practitioners who uphold the highest values of the profession including honesty and truthfulness in communication. Pretending to do otherwise despite best intention, even in the guise of a role play or OSCE, runs the risk of sending the wrong message.

## Notes On Contributors

Richard Prayson, MD, MEd is a Professor of Pathology at the Cleveland Clinic Lerner College of Medicine and Drirector of Student Affairs. He also Heads the Physican Advisory group which helps oversees the program’s portfolio assessment system.

Elizabeth O“Toole, MD is a Professor of Medicine at Case Western Reserve University School of Medicine and Director of Palliative Medicne at MetroHealth Medical Center.
