# Supplementary material for: On Truthful Introductions
Source: MedEdPublish (2016). 2018 Mar 6;7:54. [Version 1] doi: 10.15694/mep.2018.0000054.1 (PMC10712012; doi:10.15694/mep.2018.0000054.1)
Supplement: Peer review history

# Reviewer report: Reviewer response for version 1

**DOI:** 10.21956/mep.19505.r28839

**Published:** 2018-03-13

**Authors:** Julie Browne

**Affiliations:** 1 Cardiff University School of Medicine

This review has been migrated. The reviewer awarded 4 stars out of 5 This was an easy-to-read, well argued and interesting article that raised some important and topical issues. I think it is a very useful contribution to the debate around professionalism in medical students, and is relevant the current work being done on students' professional dilemmas in the clinical workplace. It is interesting that the authors had to rely on relatively old articles (20 years and more) for their underpinning data; and I do hope that this will inspire colleagues to have a go at seeing if the position has changed at all since then.

Reviewer Expertise:

NA

No decision status is available

# Reviewer report: Reviewer response for version 1

**DOI:** 10.21956/mep.19505.r28838

**Published:** 2018-03-07

**Authors:** Tan Nguyen

**Affiliations:** 1 Deakin University

This review has been migrated. The reviewer awarded 4 stars out of 5 This opinion piece was well thought out and justified. Professionalism certainly is based on trust, and reminds clinical educators and course convenors the fundamental importance to clearly define the role of the student learner.

Reviewer Expertise:

NA

No decision status is available

# Reviewer report: Reviewer response for version 1

**DOI:** 10.21956/mep.19505.r28840

**Published:** 2018-03-06

**Authors:** Trevor Gibbs

**Affiliations:** 1 AMEE

This review has been migrated. The reviewer awarded 4 stars out of 5 An interesting personal view and one that asks quite an interesting but difficult. I personally would opt for the truth, given that patients are not that easily fooled and i believe are very supportive of students in learningWill be interesting to hear others' views

Reviewer Expertise:

NA

No decision status is available
